# Supplementary material for: Disorder-Engineered Hybrid Plasmonic Cavities for Emission Control of Defects in hBN
Source: ACS Photonics. 2026 Feb 7;13(4):937–48. doi: 10.1021/acsphotonics.5c02063 (PMC12922765; doi:10.1021/acsphotonics.5c02063)
Supplement: Supplementary file 1 [file ph5c02063_si_001.pdf]

Disorder-Engineered Hybrid Plasmonic Cavities for Emission Control of Defects in hBN

Sinan Genc,[†,‡] Oguzhan Yucel,[‡,¶] Furkan Ağlarcı,[§] Carlos Rodriguez-Fernandez,[‖] Alpay Yilmaz,[‡] Humeyra Caglayan,[*,‖,⊥] Serkan Ateş,[*,§,#] and Alpan Bek[*,‡]

†*Department of Electrical and Electronics Engineering, Abdullah Gül University, 38080 Kayseri, Turkey.*

‡*Department of Physics, Middle East Technical University, 06800 Ankara, Turkey.*

¶*Department of Physics, Freie Universität Berlin, 14195 Berlin, Germany.*

§*Department of Physics, Izmir Institute of Technology, 35430 Izmir, Turkey.*

‖*Department of Physics, Tampere University, 33720 Tampere, Finland.*

⊥*Department of Electrical Engineering, Photonic Integration Group, Eindhoven University of Technology, 5600 MB Eindhoven, The Netherlands*

#*Faculty of Engineering and Natural Sciences, Sabanci University, 34956 Tuzla, Istanbul Turkey.*

E-mail: hcaglayan@tue.nl; serkan.ates@sabanciuniv.edu; bek@metu.edu.tr

Number of pages: 9

Number of figures: 7

Annealing Process

The annealing duration substantially influences the size distribution of silver nanoparticles (AgNPs) generated during dewetting. Our investigation revealed a distinct tendency of NP size augmentation with extended annealing durations. Notably, when the annealing duration was prolonged from 30 minutes to 1 hour, the average size of the AgNPs significantly increased. This results from the improved diffusion of Ag atoms across the surface over extended durations, facilitating the production of larger and more thermodynamically stable particles as presented in Figure S1.

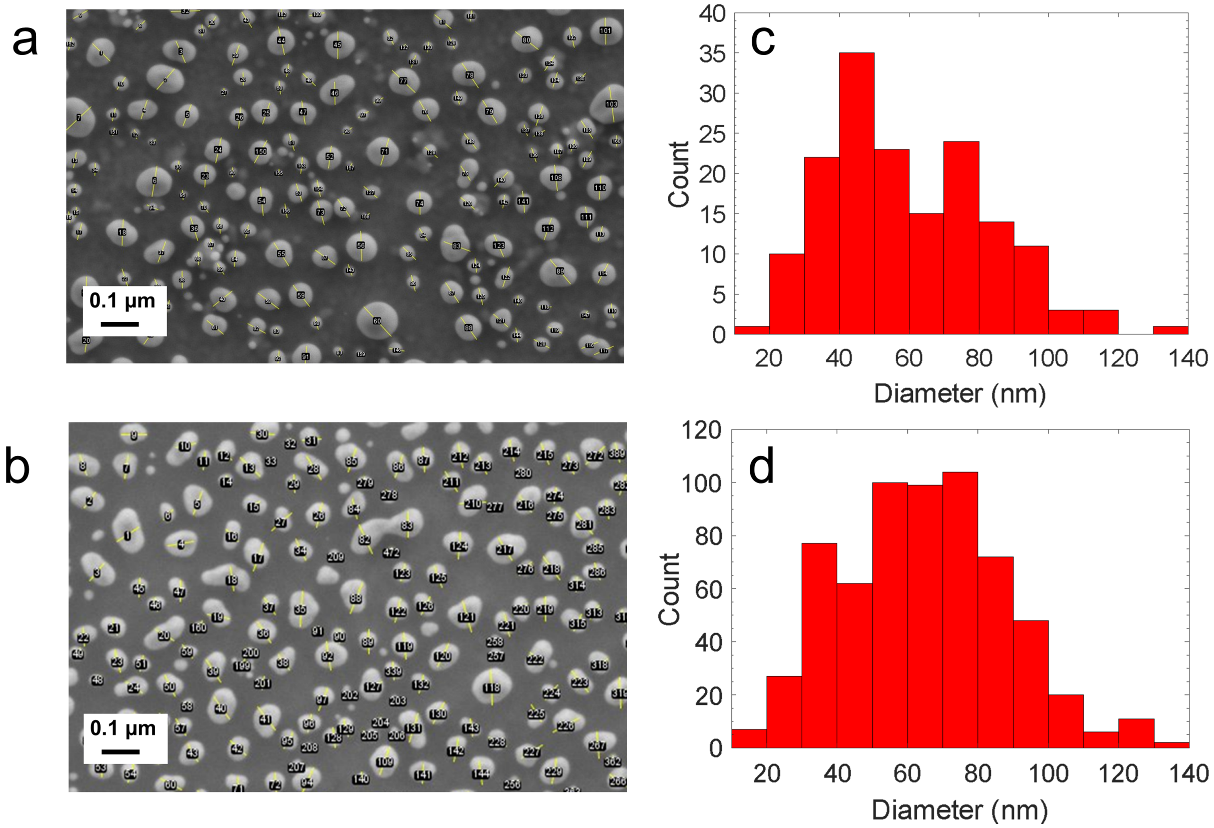

Figure S1: SEM images and grain size distribution analysis after annealing of 10 nm Ag thin film at 250°C after 30 minutes (a, c) and 1 hour (b, d), respectively.

Moreover, extended annealing durations promoted the amalgamation of smaller particles into larger formations, enhancing the observed size augmentation (Ostwald ripening). The results underscore the pivotal influence of the annealing period on the modulation of NP

size, which is crucial for enhancing plasmonic characteristics in nanophotonic applications.

FDTD Simulations

All numerical simulations were performed using a commercial finite-difference time-domain (FDTD) solver. Silver nanoparticles were modeled as hemispherical particles with diameters of 40 nm and 120 nm, as presented in Figure S2 and S3, corresponding to the average sizes extracted from SEM measurements of thermally dewetted Ag films. The optical constants of silver were taken from experimentally measured literature data. The hBN layer was modeled as a homogeneous dielectric with a refractive index of $n_{hBN} \approx 2.0$ in the visible spectral range. The emitter was represented as an oscillating electric point dipole positioned within the hBN layer at varying distances from the nanoparticle surface. Emitter–nanoparticle separations were swept from 3 to 40 nm to cover the experimentally relevant near-field coupling regime. To account for the unknown orientation of individual defect dipoles, simulations were performed for dipoles oriented parallel and orthogonal to the nanoparticle surface. The resulting spread in radiative and non-radiative decay-rate modification defines the shaded regions shown in Fig. 4d. Perfectly matched layers (PMLs) were used as boundary conditions in all directions, and a nonuniform mesh with a minimum grid size below 1 nm was employed in the near-field region to ensure numerical convergence.

The electromagnetic field distributions and scattering cross-section spectrum of the (Au-SiO_2-AgNP) structure were simulated using a commercially available Ansys Lumerical Finite-Difference Time-Domain (FDTD) software under a total-field scattered-field (TFSF) source. The design was optimized for the localization of the surface plasmon and scattering cross-section to match the possible emission of the color centers on thin hBN. Figure S6 presents Au thickness optimization simulation results. Based on these thickness-controlled scattering cross-section simulation results, an Au layer thicker than 60 nm was found to provide optimal scattering. We have opted to use a 100 nm Au layer for the experiments for ensuring the

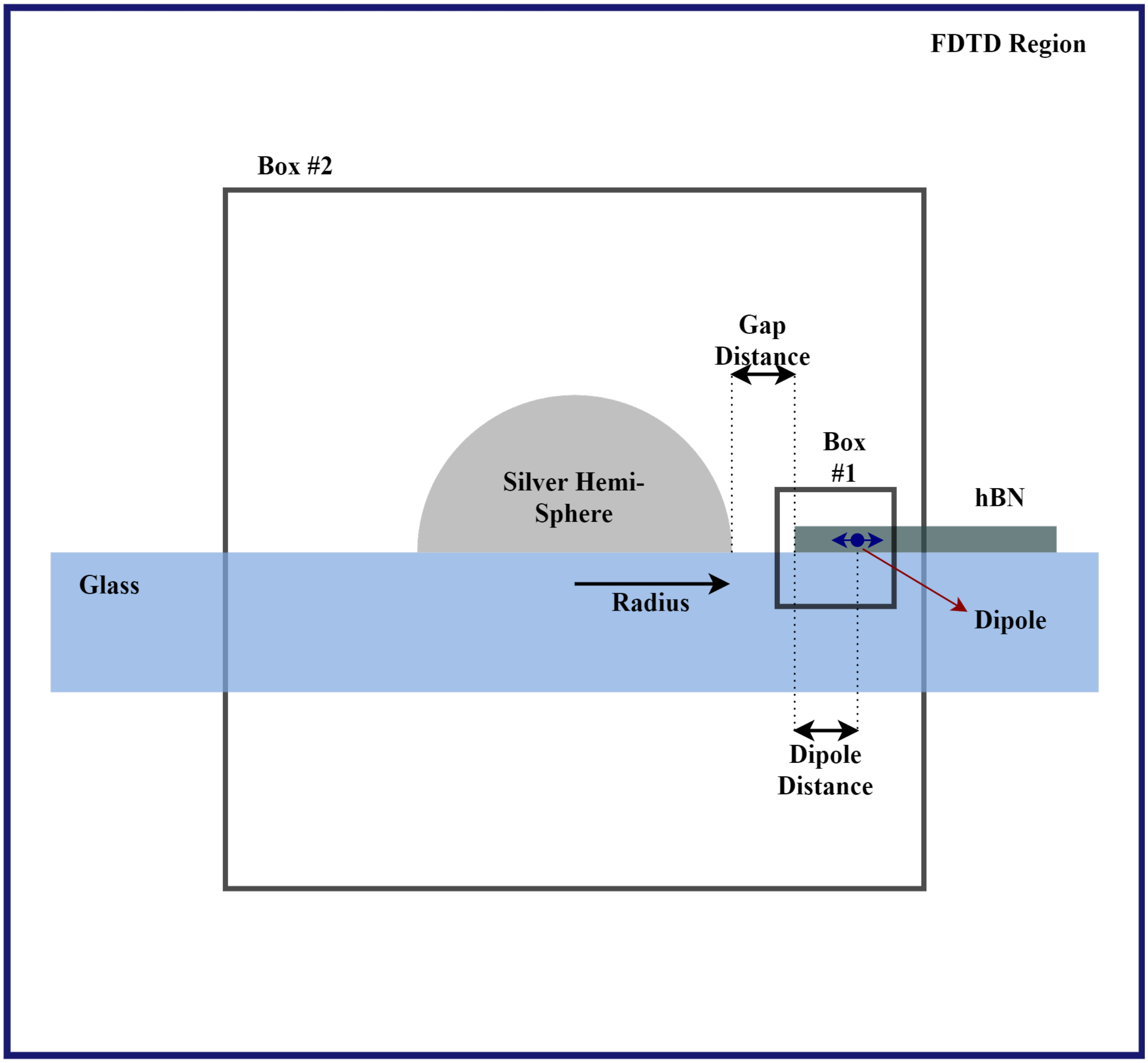

Figure S2: Computational box for the calculation of fluorescence enhancement.

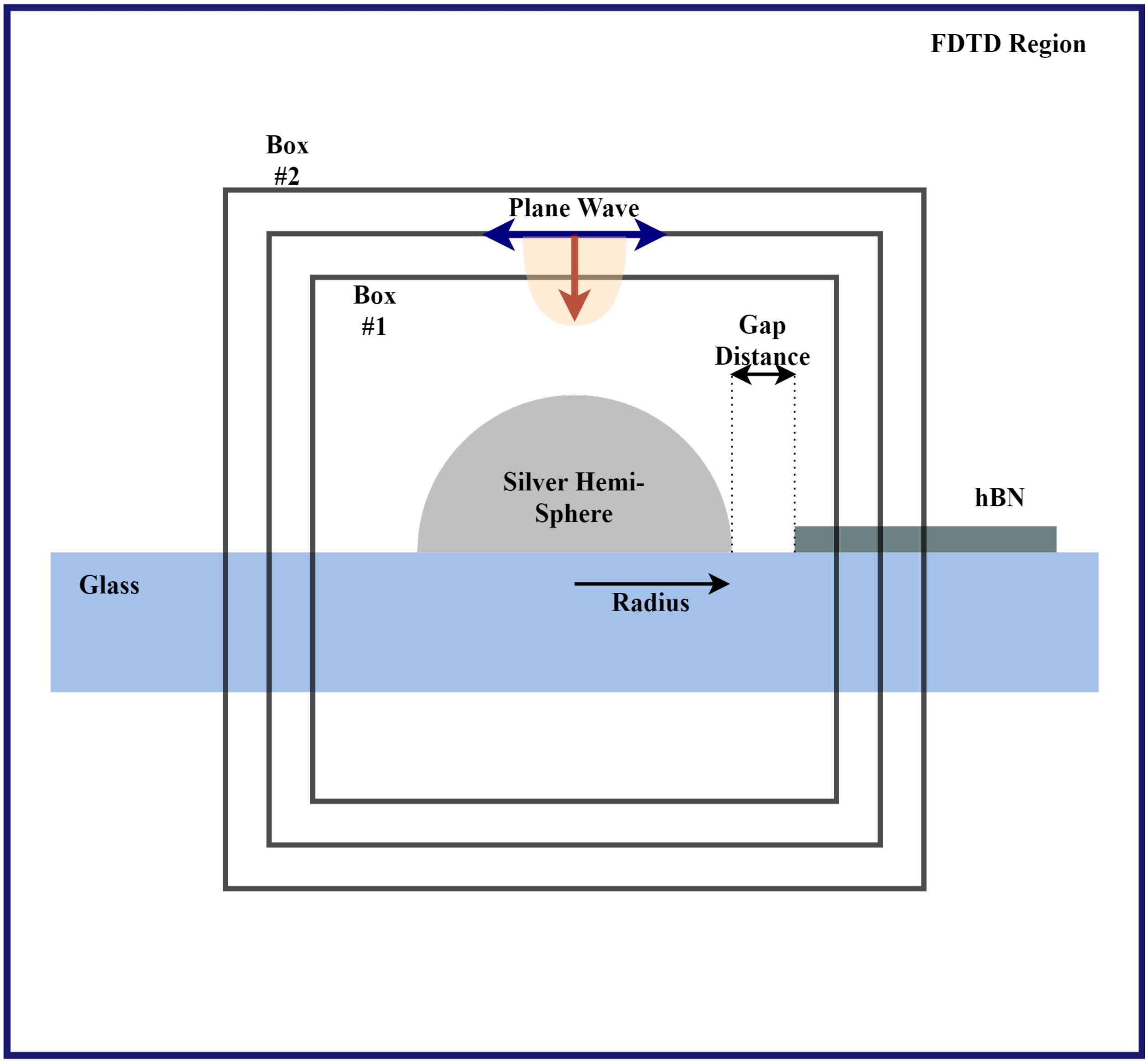

Figure S3: Computational box for the calculation of scattering cross sections.

t>60 nm. As the next optimization step, the SiO_2 spacer has been used in the range of 15 nm-35 nm. The results presented in Figure S7 reveal that 20 nm of SiO_2 provided optimal scattering within the desired wavelength range. Therefore, for the experiments, we have chosen a 100 nm Au layer, which provides the highest scattering with a spacer of 20 nm of SiO_2 using AgNPs with 70-90 nm diameter. The scattering cross sections shown in Figure S7 indicate that increasing the SiO_2 spacer thickness can enhance the scattering response for certain nanoparticle diameters. However, when the wavelength-dependent behavior is considered together with the experimentally relevant emission window (550–650 nm), a spacer thickness of 20 nm provides consistent spectral overlap with scattering-dominated plasmonic modes across all nanoparticle sizes studied.

All simulations were performed using Perfectly Matched Layer (PML) boundary conditions with an override mesh size of 0.001 µm and a simulation time of 50 fs. The Johnson–Christy model for Ag was selected from the material database.

Wavelength-Dependent Absorption and Scattering Cross Sections

To provide additional physical insight into the nanoparticle size-dependent plasmonic regimes, wavelength-dependent absorption and scattering cross sections were calculated for the two representative Ag nanoparticle diameters discussed in the main text. These results complement the decay-rate simulations by directly illustrating the relative contributions of radiative (scattering) and non-radiative (absorption) channels as a function of nanoparticle size and wavelength.

Spectral Overlap Between hBN ZPL and Nanoparticle Cross Sections

To elucidate the role of spectral overlap in plasmon–emitter coupling, wavelength-dependent absorption and scattering cross sections were calculated for the Ag nanoparticle diame-

ters investigated in the main text. These results clarify how overlap between the emitter's zero-phonon line and absorption- or scattering-dominated plasmonic responses determines whether non-radiative or radiative decay channels dominate in the strong coupling regime.

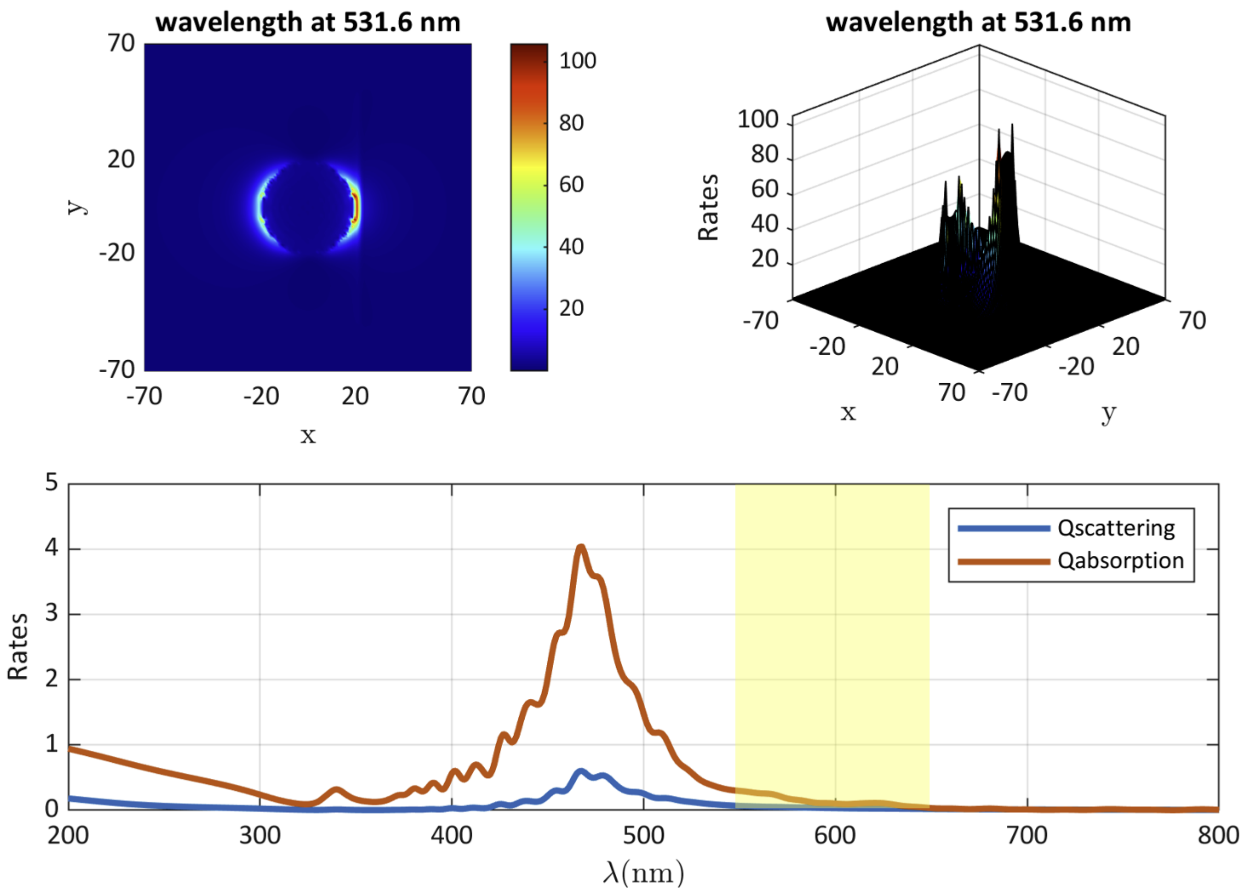

Figure S4: Wavelength-dependent absorption and scattering cross sections of a small Ag nanoparticle (diameter ∼40 nm) calculated using FDTD simulations. Absorption dominates over scattering across the spectral range relevant to hBN defect emission, explaining the experimentally observed fluorescence quenching and enhanced non-radiative decay channels (cf. Fig. 3 in the main text).

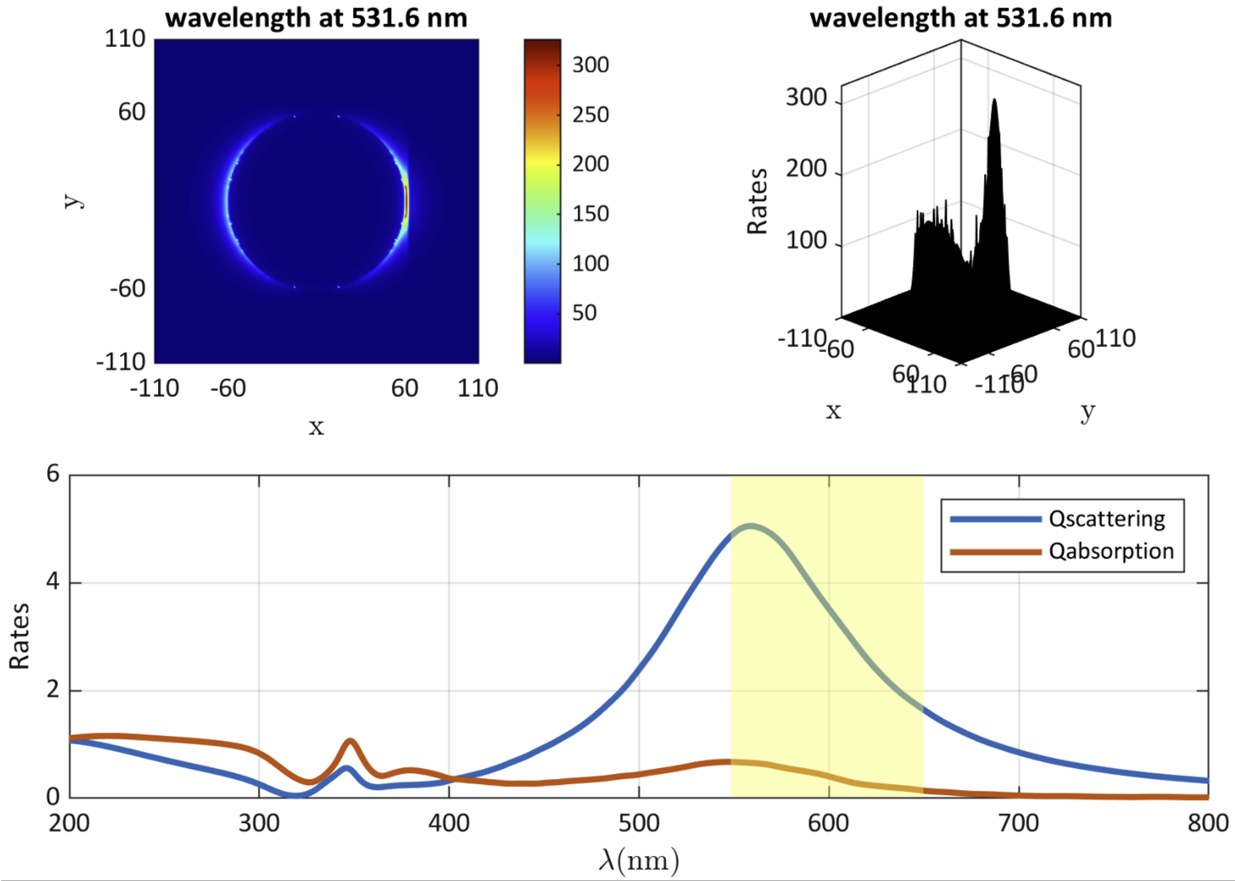

Figure S5: Wavelength-dependent absorption and scattering cross sections of a large Ag nanoparticle (diameter ∼120 nm) calculated using FDTD simulations. In contrast to smaller nanoparticles, scattering dominates over absorption in the visible spectral range, favoring radiative decay-rate enhancement and increased emission brightness, consistent with the experimental results shown in Fig. 4 of the main text.

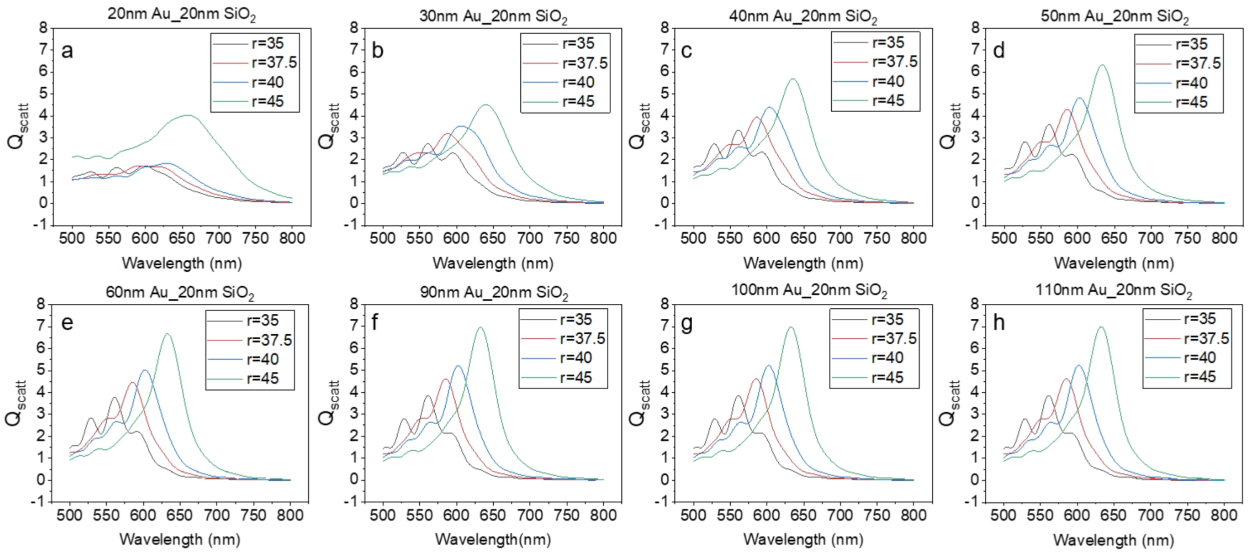

Figure S6: Scattering cross-section simulation results of different Au thicknesses for four different AgNP sizes (radius 35-45 nm) with fixed 20 nm-thin SiO_2 spacer.

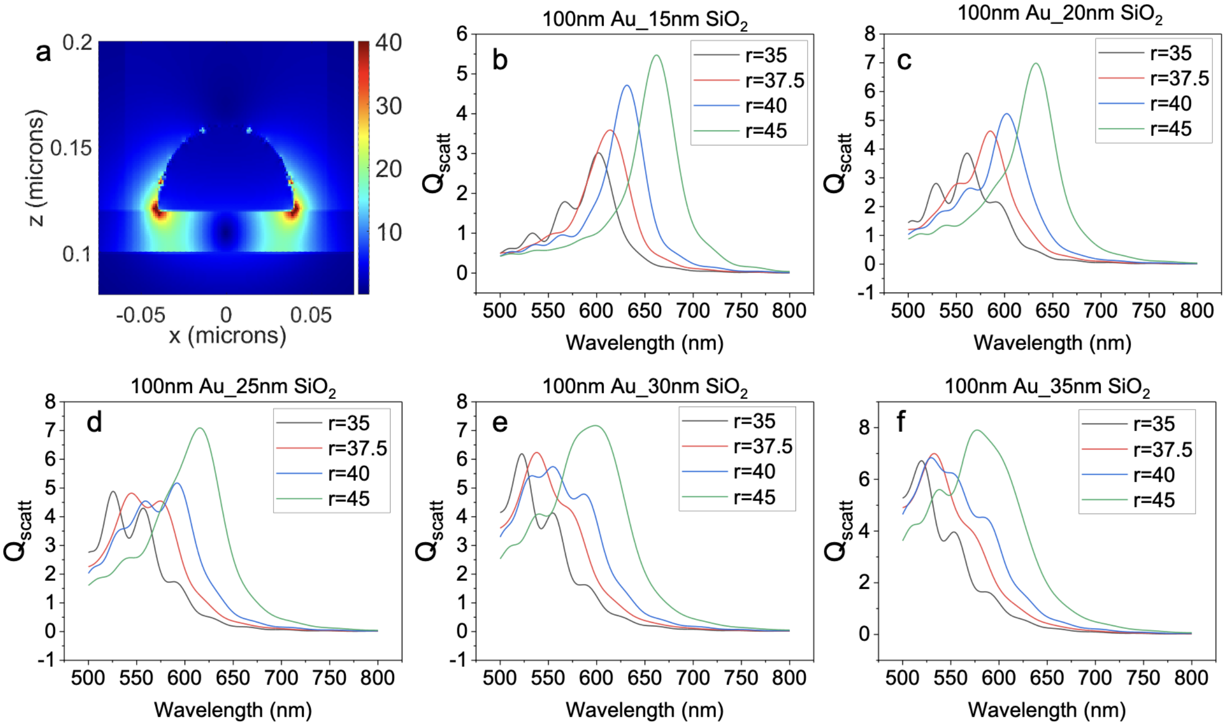

Figure S7: Wavelength-dependent scattering cross sections calculated for different SiO_2 spacer thicknesses and Ag nanoparticle diameters. While a 35 nm spacer can yield higher peak scattering cross sections for specific nanoparticle sizes (e.g., ∼90 nm), a 20 nm spacer provides consistent spectral overlap with the experimentally relevant 600–650 nm emission window across all nanoparticle diameters investigated, motivating its selection in the main experiments.
